# The GTPase-Activating Protein GRAF1 Regulates the CLIC/GEEC Endocytic Pathway

**DOI:** 10.1016/j.cub.2008.10.044

**Published:** 2008-11-25

**Authors:** Richard Lundmark, Gary J. Doherty, Mark T. Howes, Katia Cortese, Yvonne Vallis, Robert G. Parton, Harvey T. McMahon

**Affiliations:** 1 Medical Research Council, Laboratory of Molecular Biology, Hills Road, Cambridge, CB2 0QH, UK; 2 Institute for Molecular Bioscience and Centre for Microscopy and Microanalysis, University of Queensland, Brisbane, Queensland 4072, Australia

**Keywords:** CELLBIO

## Abstract

Clathrin-independent endocytosis is an umbrella term for a variety of endocytic pathways that internalize numerous cargoes independently of the canonical coat protein Clathrin [1, 2]. Electron-microscopy studies have defined the pleiomorphic *CL*athrin-*I*ndependent *C*arriers (CLICs) and *G*PI-*E*nriched *E*ndocytic *C*ompartments (GEECs) as related major players in such uptake [3, 4]. This CLIC/GEEC pathway relies upon cellular signaling and activation through small G proteins, but mechanistic insight into the biogenesis of its tubular and tubulovesicular carriers is lacking. Here we show that the Rho-GAP-domain-containing protein GRAF1 marks, and is indispensable for, a major Clathrin-independent endocytic pathway. This pathway is characterized by its ability to internalize bacterial exotoxins, GPI-linked proteins, and extracellular fluid. We show that GRAF1 localizes to PtdIns(4,5)P2-enriched, tubular, and punctate lipid structures via N-terminal BAR and PH domains. These membrane carriers are relatively devoid of caveolin1 and flotillin1 but are associated with activity of the small G protein Cdc42. This study provides the first specific noncargo marker for CLIC/GEEC endocytic membranes and demonstrates how GRAF1 can coordinate small G protein signaling and membrane remodeling to facilitate internalization of CLIC/GEEC pathway cargoes.

## Results and Discussion

**Figure 1:**
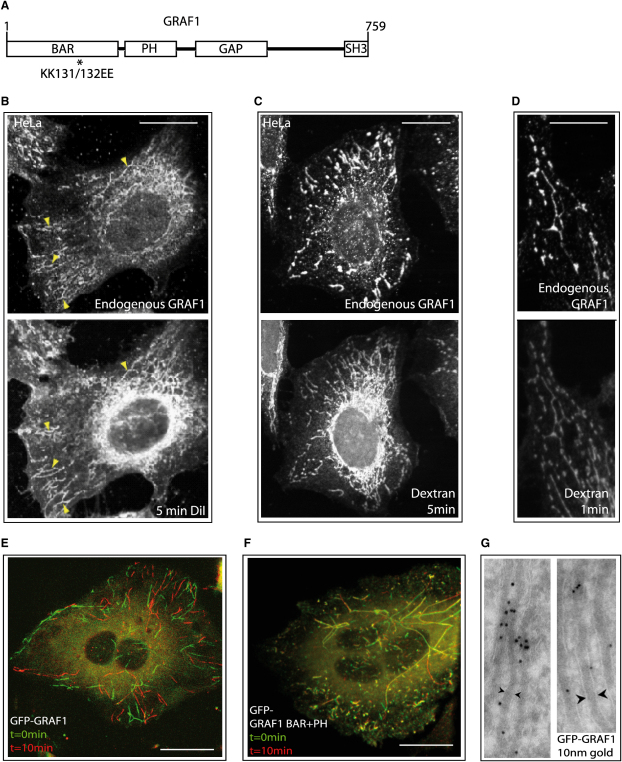
GRAF1 Tubules Are Highly Dynamic and Mark a Prevalent Endocytic Pathway (A) Domain architecture of GRAF1 and the site of introduced BAR domain mutations (^∗^). (B–D) Micrographs showing that GRAF1-positive tubules are derived from the plasma membrane as shown by colabeling with the membrane dye DiI after 5 min (B) or internalized dextran after either 5 min (C) or 1 min (D) of incubation. (E) Overlaid maximum projections of spinning-disc confocal micrographs of HeLa cells expressing GFP-tagged GRAF1 demonstrate that GRAF1-positive tubules are completely turned over in 10 min. Z sections were performed continuously for 10 min in 0.5 μm steps. The initial maximum projection (in green) was then merged with the final maximum projection (in red). See also Movie S1. (F) Overlaid maximum projections as in (a), but for a cell overexpressing GFP-tagged GRAF1 BAR+PH. See also Movie S2. (G) Electron micrographs prepared as described and immunolabeled for GRAF1 (10 nm gold particles). Note the GRAF1-positive tubular structures. Distance between arrowhead tips = 40 nm. Scale bars represent 10 μm.

The protein *G*TPase *R*egulator *A*ssociated with *F*ocal Adhesion Kinase-1 (GRAF1) is predicted to comprise an N-terminal BAR domain, a PH domain, a RhoGAP domain, a proline-rich domain, and a C-terminal SH3 domain (Figure 1A). GRAF1 exhibits GAP activity for the small G proteins RhoA and Cdc42 and has been shown to interact with the kinases FAK and PKNβ [5, 6, 14]. The presence of a predicted BAR domain in GRAF1 suggests that it might function in membrane sculpting [7]. GRAF1 was found to be expressed in a variety of cell lines (Figure S1A), and by immunocytochemistry we found that GRAF1 was predominantly localized to pleiomorphic tubular and punctate structures in HeLa and NIH 3T3 cells (Figures 1B–1D and Figure S1B). Although GRAF1 can be found primarily on long tubules in some cells, other cells within the same population exhibit a predominantly punctate GRAF1 localization. GRAF1-positive tubules are disrupted at low temperatures, and a 37°C fixation was required for their integrity to be preserved (Figure S1C). Upon monitoring both GRAF1 localization and endocytosis of either the plasma membrane marker DiI or the fluid-phase marker dextran by confocal microscopy, we found that endocytic structures extensively colocalized with GRAF1 (Figures 1B and 1C). Colocalization of GRAF1 was even evident when only very early endocytic structures were examined, indicative of an early endocytic role for GRAF1-positive membranes (Figure 1D). We then examined the turnover of GRAF1-positive membranes with time by overexpressing GFP-tagged full-length GRAF1 in HeLa cells and examining its localization by using four-dimensional spinning-disc confocal microscopy. We found GRAF1-positive tubular structures to be spectacularly dynamic (Figure 1E and Movie S1). By electron microscopy, we found that GRAF1-labeled tubules were around 40 nm in diameter in vivo (Figure 1G). We also found that overexpressed GFP-tagged GRAF1 BAR+PH protein (missing the GAP, proline-rich, and SH3 domains) also labeled tubular membranes that could accumulate DiI (Figure 1F, Movie S2, and data not shown). However, these tubules were much more static than those labeled by overexpressed full-length GRAF1, suggesting that GRAF1 BAR+PH might act in a dominant-negative manner to stabilize early endocytic tubules (see also below).

**Figure 2:**
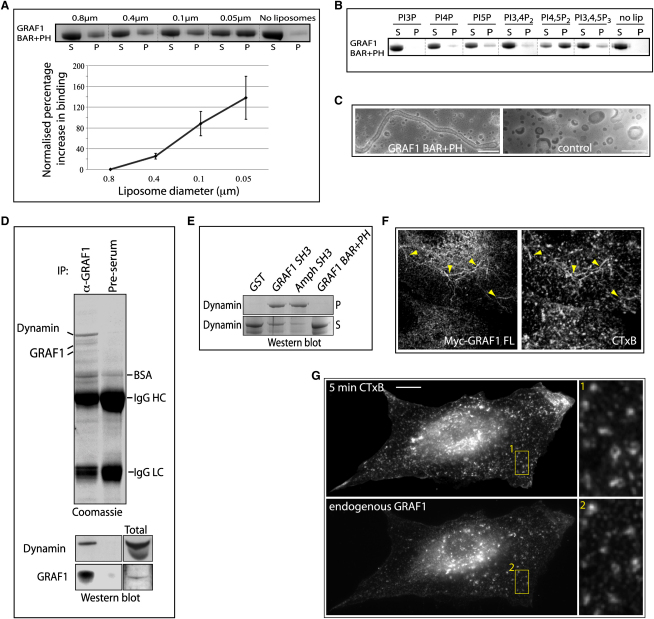
The BAR and PH Domains Localize GRAF1 to Highly Curved, PtdIns(4,5)P2-Enriched Membranes, and the SH3 Domain Binds Dynamin (A) Coomassie-stained gel of liposome cosedimentation assay showing the preference of GST-tagged GRAF1 BAR+PH protein for binding to smaller-sized liposomes derived from total brain lipids (the average diameters of liposomes are shown). Pellet (P) and supernatant (S) fractions were separated by ultracentrifugation. The graph shows quantifications of total band intensities for each condition normalized to binding of the non-curvature-sensitive protein Dab2 (as a way of controlling for total lipid in each experiment). The error bars show 95% confidence intervals (calculated by t tests) for each condition. (B) Liposome cosedimentation assay as performed in (A) but with 0.8-μm-diameter liposomes enriched with varying phosphoinositides. (C) Electron micrographs of negatively stained liposomes incubated in the presence or absence of GST-tagged GRAF1 BAR+PH protein. Note the protein-dependent presence of tubular structures. The scale bar represents 200 nm. (D) Immunoprecipitation of GRAF1 from rat brain cytosol (via Ab2) reveals a GRAF1-Dynamin1 complex as identified by mass spectrometry and confirmed by immunoblot. (E) Coomassie-stained gels of pull-down experiments with purified Dynamin and either bead-bound GST-tagged GRAF1/Amphiphysin SH3 domain or GRAF1 BAR+PH protein. P = pellet fraction, S = supernatant fraction. (F and G) Epifluorescence micrographs of HeLa cell overexpressing Myc-tagged GRAF1 and incubated with CTxB for 5 min before fixation and staining. (G) Epifluorescence micrographs of a HeLa cell incubated with CTxB for 5 min before fixation and staining for endogenous GRAF1. Scale bars represent 10 μm.

To examine the specific properties of the predicted lipid-binding region of GRAF1, we performed lipid cosedimentation assays with purified GRAF1 BAR+PH protein (Figure 2A). Using liposomes of varying diameter, we found that GRAF1 BAR+PH bound better to smaller (more highly curved) liposomes, consistent with the presence of a membrane-curvature-sensing BAR domain [7]. Furthermore, we examined the effects of mutating key lysine residues [7] in the BAR domain to glutamates (KK131/132EE) and found that this mutant was now cytoplasmically distributed (Figure S1D). Expression of the BAR domain alone in cells resulted in a predominantly cytoplasmic and sometimes punctate localization (Figure S1E), suggesting that both the BAR and PH domains are necessary for tubular localization of GRAF1. We therefore tested whether, in addition to a curvature-sensing or -generating capability, the GRAF1 BAR+PH unit has phosphoinositide-binding specificity. GRAF1 BAR+PH protein was subjected to lipid cosedimentation assays with 10% phosphatidylserine-containing liposomes with varying phosphoinositide composition (Figure 2B). Greatest binding was observed for PtdIns(4,5)P2-enriched liposomes. PtdIns(4,5)P2 is plasma-membrane enriched [8], consistent with our observations that GRAF1-associated trafficking occurs from this site. GRAF1 BAR+PH protein was also capable of generating tubules in vitro from spherical liposomes as examined by electron microscopy under negative-staining conditions (Figure 2C). The diameter of these tubules was around 40 nm, consistent with the observed diameter of GRAF1-positive tubules found in cells (Figure 1G). Taken together, these data strongly suggest that the BAR and PH domains of GRAF1 function together to produce and/or stabilize endocytic tubules in vivo.

To identify interacting partners for GRAF1, we immunoprecipitated GRAF1 from rat brain cytosol. Interestingly, GRAF1 was found in a complex with the membrane scission protein Dynamin1 (Figure 2D and S2A). We confirmed binding to both Dynamin1 and Dynamin2 by using GST-tagged GRAF1 SH3 domain as bait in pull-down experiments against purified Dynamin1, brain cytosol, or HeLa cell lysate (Figure 2E; also Figures S2B and S2C). Monomeric GRAF1 SH3 domain was found to bind to a peptide from Dynamin1 proline-rich domain with a k_d_ of 106 μM (Figure S2D). Furthermore, treatment of HeLa cells with dynasore resulted in a profound reduction of tubular endocytosis in HeLa cells and a redistribution of GRAF1 to basally located puncta (Figure S2E). These data, taken together with our observation that C-terminally truncated versions of GRAF1 (lacking the SH3 domain) are found on long static tubules, suggests that the complex between Dynamin and GRAF1 might function to regulate the scission and stability of these ordinarily pleiomorphic tubulovesicular structures.

Clathrin polymers are rarely found on tubular membranes by electron microscopical techniques. Indeed, GRAF1-positive tubules, and other GRAF1-positive structures, were devoid of Clathrin and did not colocalize with internalized or internalizing transferrin or the transferrin receptor, which is widely used as a marker for Clathrin-mediated endocytic events (Figures S3A–S3C). Alongside the canonical Clathrin-mediated endocytic routes, other prevalent endocytic pathways coexist [1, 2]. For example, the internalization of MHC class I proteins, many GPI-linked receptors, and bacterial exotoxins rely on trafficking compartments with heretofore undefined coat components. The data presented above strongly suggest that GRAF1 might function as a coat within a prevalent Clathrin-independent endocytic pathway. To characterize further the nature of GRAF1-positive tubules, we incubated GRAF1-overexpressing HeLa cells with cholera toxin B subunit (CTxB), a marker that is used for the study of Clathrin-independent endocytic pathways but that also enters via Clathrin-mediated routes [9]. We found that internalized CTxB colocalized with Myc-GRAF1-positive tubules in HeLa cells (Figure 2F) as well as with endogenous GRAF1-positive structures after 5 min incubation at 37°C (Figure 2G). In addition, we found that GRAF1 BAR+PH overexpression (which leads to static tubules; Figure 1F) halved the number of cells internalizing CTxB without effecting transferrin uptake (Figures S4A–S4C). We then followed CTxB and transferrin internalization in real time in NIH 3T3 cells (almost all of which are capable of binding and internalizing CTxB; cf. HeLa cells, which often lack its glycolipid receptor, GM1). NIH 3T3 cells were transfected with constructs encoding either GFP-tagged GRAF1 or GRAF1 BAR+PH and incubated with CTxB and transferrin on ice before stimulation of endocytosis by incubation of cells with prewarmed (37°C) media. Imaging commenced immediately (Figure S4D and Movie S3). Newly forming dynamic GRAF1-positive tubules were shown to contain CTxB at extremely early stages of its internalization and were unassociated with internalizing/internalized transferrin. Furthermore, the more static, elongated GRAF1 BAR+PH-positive tubules maintained the presence of CTxB, again independently of transferrin, suggesting that here the toxin is trapped in structures that cannot undergo fission from the plasma membrane and progress further (data not shown).

Although GRAF1-positive carriers resemble the previously characterized Arf6-dependent structures that are responsible for uptake of MHC Class I proteins [10], we found no evidence for overlap between these pathways. Neither GRAF1- nor GRAF1 BAR+PH-positive tubules were found to contain endocytosed or recycled MHC class I proteins at 5, 15, or 45 min after stimulation of their internalization when specific antibodies were used, and the amount of total MHC class I protein endocytosis was not altered upon overexpression of the dominant-negative GRAF1 BAR+PH construct (Figures S6A and S6B and data not shown). In addition, the tubular localization of GRAF1 was independent of overexpression of wild-type Arf6 or dominant-active Arf6 (Arf6Q67L; data not shown). In contrast, Arf6 Q67L overexpression was found to completely block the internalization of MHC class I molecules ([11] and data not shown).

**Figure 3:**
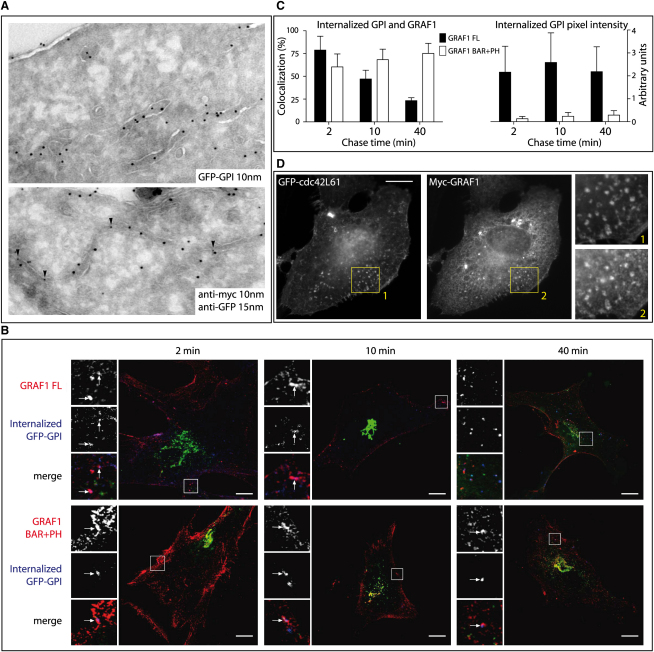
GRAF1 Regulates the CLIC Endocytic Pathway (A) Representative electron micrographs of 65 nm ultrathin cryosections of HeLa cells transiently transfected with GFP-GPI alone or with both GFP-GPI and Myc-tagged GRAF1 FL. Cells were fixed in 2% PFA with 0.2% glutaraldehyde and labeled with anti-GFP and anti-Myc antibodies. Protein A 10 nm gold was used for revealing the GFP in the single labeling (upper image). As shown, GFP-GPI was found in uncoated vesicles and tubules within the cell. Double labeling of GRAF1 (Protein A gold 10 nm) and GFP-GPI (Protein A gold 15 nm) is shown in the bottom image, where colocalization of GRAF1 and GFP-GPI is seen in an intracellular tubule. Arrowheads point to 10 nm gold particles. Due to limitations of the Tokuyasu method, it is not possible to discriminate cell surface-connected tubules from intracellular tubules. (B) Caveolin1 knockout MEFs were cotransfected with GFP-GPI (green) and either Myc-tagged GRAF1 (upper row; red) or GRAF1 BAR+PH (lower row; red). GFP antibodies (blue) were bound to cells for 30 min on ice prior to induction of internalization at 37°C and 5% CO_2_ for the indicated times. Surface labeling was removed, and cytosol extraction followed (see Supplemental Experimental Procedures). Panels on the left side of each image show GRAF1 proteins (top), internalized anti-GFP (middle), and a merged, triple-labeled image (bottom). Arrows indicate points of colocalization. (C) Micrographs of Caveolin1 knockout MEFs co-overexpressing GRAF1 and GFP-GPI, after anti-GFP internalization, acid stripping, and cytosol extraction, were processed with Volocity 3.7.0 so that the percentage colocalization could be calculated. The histogram shows the number of anti-GFP pixels that colocalize with GRAF1 relative to all anti-GFP pixels. Five to seven images across three independent experiments were taken after 2, 10, and 40 min of internalization. Error bars indicate the standard errors of the mean. Images were also processed in Adobe Photoshop CS2 so that the number of anti-GFP pixels relative to total pixels within the image could be calculated. The histogram represents standardized values of anti-GFP pixels. Error bars indicate the standard errors of the mean. (D) Confocal micrographs of HeLa cells co-overexpressing GFP-tagged Cdc42 L61 (dominant-active) and Myc-tagged GRAF1 showing colocalization in discrete puncta and short tubules. Scale bars represent 10 μm.

Electron-microscopy studies, focusing on the uptake of CTxB and GPI-linked receptors, have suggested that a large portion of these proteins are internalized by *Cl*athrin-*i*ndependent *c*arriers (CLICs) seemingly into *G*PI-AP-enriched *e*arly *e*ndosomal *c*ompartments (GEECs), together with fluid-phase markers such as dextran [4]. Our data have shown that GRAF1-positive structures morphologically and functionally resemble the endocytic structures of this CLIC/GEEC endocytic pathway [4]. We therefore examined whether GRAF1 could regulate the internalization of a model GPI-linked protein known to undergo endocytosis into the CLIC/GEEC pathway. Consistent with this, we found by using immuno-electron microscopy that GRAF1 labeled GFP-GPI-positive tubules (Figure 3A). We then examined the internalization of GFP-GPI in mouse embryonic fibroblasts by binding anti-GFP antibodies and transferrin (as a control) to cells on ice and subsequently moving these cells to 37°C for variable chase periods. We did this in both wild-type (data not shown) and caveolin1-knockout mouse embryonic fibroblasts (to exclude caveolae-associated uptake) that we transfected with either a Myc-tagged GRAF1 or a Myc-tagged GRAF1 BAR+PH construct and examined after cytosol washout (Figure 3B; note that washout treatment damages GRAF1-positive membranes but is necessary for the objective quantitative analysis of colocalization of GRAF1 with internalized GFP-GPI because cytoplasmic GRAF1 would otherwise result in overestimation of colocalization). GRAF1 was found to significantly colocalize with internalized GFP-GPI (79% colocalization) but not transferrin after a 2 min chase (Figures 3B and 3C and data not shown). A reduced level of colocalization was seen after a 10 min chase (47%), and even lower colocalization levels were observed after a 40 min chase (when the protein is recycling back to the plasma membrane; 23%). These data are consistent with our previous observations, strongly suggesting a role for GRAF1 function in sculpting the highly curved membranes of the CLIC/GEEC endocytic pathway. In contrast, GRAF1 BAR+PH consistently colocalized with GFP-GPI after 2 min (60%), 10 min (68%), and 40 min (75%) chase periods, further supporting a role for this protein as a dominant-negative protein that traps early CLIC/GEEC carriers. To characterize this dominant-negative effect in greater detail, we examined the effect of GRAF1 BAR+PH protein on GFP-GPI internalization (Figures 3B and 3C). Compared with GFP-GPI internalization in the presence of GRAF1, the total amount of GFP-GPI internalization was profoundly reduced in the presence of GRAF1 BAR+PH (to around 10% of control levels), suggesting further that this protein traps cargoes in rather static early endocytic carriers that are incapable of undergoing fission from the plasma membrane. Importantly, the steady-state surface levels of GFP-GPI were indistinguishable between cells expressing GRAF1 and those expressing GRAF1 BAR+PH (Figure S6C).

CTxB has been shown to be endocytosed via caveolin and flotillin-positive structures [12]. Although we did not find caveolin1 or flotillin1 in GRAF1-positive structures at steady state by fluorescence microscopy (Figures S5A and S5B), stabilization of early GRAF1-positive carriers by GRAF1 BAR+PH overexpression caused flotillin1, and to a lesser extent caveolin1, to be found in such regions with CTxB (Figures S5C and S5D), suggesting that these membrane regions can communicate with the CLIC/GEEC pathway.

Uptake through the CLIC/GEEC endocytic pathway has previously been shown to be Cdc42 dependent [3]. Consistent with this, we observed that overexpressed dominant-active Cdc42 protein colocalizes with GRAF1 in basally located puncta (Figure 3D). The absence of Cdc42 in long tubular structures might suggest that the role of Cdc42 in this pathway is restricted spatially and temporarily to the earlier endocytic stages or that progression is blocked through the dominant-active construct, which would be expected to have pleiotropic effects.

**Figure 4:**
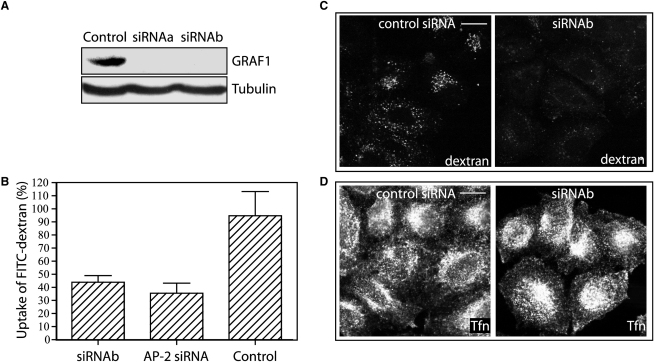
GRAF1 Is Indispensable for Clathrin-Independent Fluid-Phase Uptake in Fibroblasts (A) Immunoblots on HeLa cell lysates transfected with a control siRNA or either of two siRNAs directed against GRAF1 mRNA (siRNAs a and b). Detection of GRAF1 and tubulin (loading control) was performed with specific antibodies on lysates obtained 48 hr after transfection. (B) GRAF1-depleted cells show a major reduction in fluid-phase endocytosis as shown by the decrease in the uptake of FITC-labeled dextran (control siRNA (n = 5), siRNAa (n = 8), or AP2 siRNA (n = 3)). The error bars show the standard deviation of the mean. (C and D) HeLa cells depleted of GRAF1 were incubated with dextran (C) or transferrin (D) for 15 min before fixation and staining. Scale bars represent 10 μm.

To determine whether GRAF1 was necessary for endocytosis, we depleted GRAF1 levels by using siRNA. This treatment was capable of reducing the amount of GRAF1 to background levels as assessed by immunocytochemistry and immunoblotting of HeLa cells and their lysates, respectively (Figure 4A; also Figure S6D). GRAF1-depleted cells were assessed for their ability to endocytose dextran (allowing assessment of total endocytic capacity), both by epifluorescence microscopy and by a quantitative fluorimetric assay (Figures 4B and 4C). GRAF1 depletion resulted in a 50%–60% reduction of total dextran endocytosis, similar to that observed upon depletion of AP2, the major Clathrin adaptor at the plasma membrane (Figure 4B), suggesting that GRAF1-mediated endocytosis and Clathrin-mediated endocytosis account for roughly equal amounts of volume internalization in these cells. GRAF1-depleted cells had no observable defects in their ability to endocytose transferrin (Figure 4D), demonstrating that GRAF1 is not necessary for Clathrin-mediated endocytosis.

In this study we have shown that GRAF1 is found on tubular and vesicular membranes in vivo, and that these membranes define a prevalent Clathrin-independent endocytic pathway. This endocytic pathway is capable of internalizing Cholera toxin, GPI-linked proteins, and large amounts of extracellular fluid, and it corresponds (at least in part) to the CLIC/GEEC endocytic pathway. Activity of Arf1, in addition to that of its GEF ARHGAP10, is necessary for uptake via the CLIC/GEEC pathway, and Arf1 activity modulation also interferes with Cdc42 dynamics, suggesting interplay between these proteins [13]. Indeed, GRAF1 is capable of downregulating the activity of Cdc42 [14], which is known to be necessary for the CLIC/GEEC pathway [3] and which colocalizes with GRAF1 in vivo. The protein machinery responsible for the biogenesis and processing of CLIC/GEEC endocytic membranes is largely unknown. Although Dynamin certainly plays a role in CLIC/GEEC membrane processing (it is necessary for delivery of CTxB to the Golgi apparatus [4]), its precise role in the CLIC/GEEC pathway is uncertain. Recent microinjection experiments with antibodies directed against specific isoforms of Dynamin, or with siRNAs directed against these, showed that Dynamin is involved in constitutive fluid-phase uptake and suggested that it might play an important role in high-volume Clathrin-independent endocytic events [15]. We build on this work by showing that there exists a tight biochemical interaction between GRAF1 and Dynamin. Although we implicate Dynamin in this pathway, precisely determining whether it functions here in an analogous manner to Dynamin at Clathrin-coated pits or works via a noncanonical mechanism will require further work.

Our experiments lead us to suggest that GRAF1 coordinates the highest volume Clathrin-independent endocytic pathway in HeLa cells. Although several Clathrin-independent endocytic pathways appear to coexist (such pathways include caveolin1- and flotillin1-positive pathways, an Arf6-associated tubular uptake pathway, and the CLIC/GEEC pathway), the precise contributions that each of these pathways makes to constitutive endocytosis, and the interrelationships between these pathways, are uncertain. Although we have found that GRAF1-positive membranes contain little flotillin1 or caveolin1 at steady state, we have also shown that these membranes probably transiently communicate. We further show that GRAF1-positive membranes are not associated with internalizing or recycled MHC class I proteins. Taken together, our data link biochemical and cell biological observations to identify the first important modulator of membrane curvature in a highly prevalent Clathrin-independent endocytic pathway. We believe that, in the context of our findings, study of Clathrin-independent endocytic pathways will reveal new layers of complexity underlying lipid and protein cargo selection, appropriate intracellular trafficking of endocytic membranes, and the mechanisms of endocytic intermediate formation by membrane deformation and membrane-curvature stabilization.

## Supplemental Data

Document S1. Supplemental Experimental Procedures and Six Figures

Movie S1. GRAF1-Positive Tubules Are Highly Dynamic Movie of HeLa cell overexpressing GFP-tagged GRAF1, manipulations of which are shown in Figures 2A and 2B. Movie speed is 40 times real time.

Movie S2. GRAF1 BAR+PH-Positive Tubules Are Relatively Static Movie of HeLa cell overexpressing GFP-tagged GRAF1 BAR+PH, a manipulation of which is shown in Figure 2D. Movie speed is 40 times real time.

Movies S3. CTxB Internalization Occurs into GRAF1-Positive Tubules Movie of NIH 3T3 cells overexpressing GFP-tagged GRAF1 (stills of which are shown in Figure 6D) and incubated with CTxB as described. Movie speed is 80 times real time.
