# Supplementary material for: The GTPase-Activating Protein GRAF1 Regulates the CLIC/GEEC Endocytic Pathway
Source: Curr Biol. 2008 Nov 25;18(22-2):1802–8. doi: 10.1016/j.cub.2008.10.044 (PMC2726289; doi:10.1016/j.cub.2008.10.044)
Supplement: Document S1. Supplemental Experimental Procedures and Six Figures [file mmc1.pdf]

Supplemental Data

The GTPase-Activating Protein GRAF1 Regulates the CLIC/GEEC Endocytic Pathway

Richard Lundmark, Gary J. Doherty, Mark T. Howes, Katia Cortese, Yvonne Vallis, Robert G. Parton, and Harvey T. McMahon

Supplemental Experimental Procedures

cDNA construct preparation

cDNA constructs encoding human GRAF1 (amino acids 1-759), GRAF1-BAR+PH (amino acids 1-383), GRAF1 PH+GAP (amino acids 267-576), and GRAF1-SH3 (694-759) were amplified from IMAGE clone 30343863 using PCR and cloned into the pGEX- 4T-2 vector for bacterial expression (Amersham Biosciences). GRAF2 SH3 domain was amplified similarly from IMAGE clone 6188298 and cloned into the pGEX-4T-2 vector. Fragments were also cloned into the pCMVmyc vector with added Not1 site (a kind gift from JGW Anderson) or EGFP-C3 (Clontech) for mammalian expression. Amino acid substitutions K131E, K132E and R412D were created using PCR directed mutagenesis (Stratagene). The GFP-tagged Cdc42 L61 consrtruct was a kind gift from M. Fällman. pEGFP-GPI was a generous gift from S. Mayor. caveolin1-GFP and flotillin1-GFP were kind gifts from Ben Nichols.

Protein expression, protein purification and antibodies

Recombinant proteins were expressed in a BL21 (DE3) pLysS *E-coli* strain as Glutathione S-transferase (GST)- fusion proteins and purified using glutathione-Sepharose 4B beads (Amersham Biosciences) and gel filtration on a sephacryl S-200 column (Amersham) as previously described [1]. Polyclonal antisera against GRAF1 were generated by immunising Rabbits (Ra83/Ab1 directed against the SH3 domain; Ra84/Ab2 directed against the PH+GAP domains; and RaZ1/Ab3 directed against the full length protein; all of these were used for Western blotting analyses, and Ab1/3 also used for immunofluorescence analyses) and a chicken (CH-9798/Ab4, directed against the full length protein, used for immunofluorescence analyses) with recombinantly expressed human GRAF1 proteins. Purchased antibodies were: mouse anti-myc clone 9E10, mouse anti-tubulin (Sigma-Aldrich), Rabbit anti-myc (Cell Signalling Technology), mouse anti-Dynamin, (BD Transduction Laboratories), Rabbit anti-synaptojanin Ra59 [1], (Affinity Bioreagents). All secondary antibodies and streptavidins were conjugated to Alexa Fluor 488, 546 or 647 (Invitrogen).

Expression, immunoprecipitation and pull down experiments

For analysis of endogenous protein expression, cell lines were grown according to instructions from American Tissue Culture Collection, harvested and lysed in 1% NP-40 in PBS supplemented with protease inhibitors. After a 20,000g centrifugation the supernatant was analysed by SDS-PAGE and immunoblotting. For immunoprecipitation experiments, rat brain cytosol was generated by homogenization of rat brains in buffer (25mM HEPES, 150mM NaCl, 1mM DTT, 0.1% Triton X-100 and protease inhibitors), before centrifugation at 50,000rpm for 30 minutes at 4°C. The supernatant was removed and added to protein A Sephorose 4B beads (Amersham Biosciensis) to which antibodies had been previously bound and incubated at 4°C for 3 hours. Beads were washed three times in buffer (25mM HEPES, 150mM NaCl) supplemented

with 1% NP-40, and once in buffer without NP-40 before analysis by SDS-PAGE combined with immunoblotting or Coomassie staining. Pull-down experiments against rat brain cytosol using purified proteins and identification by mass-spectrometry were performed as previously described [1].

Protein and lipid interaction assays

Liposomes from total brain lipids (FOLCH fraction I) (Sigma Aldrich) or synthetic lipids (Avanti Polar Lipids), and liposomes of a specified diameter or phosphoinositide enrichment were generated as previously described [2]. Liposome binding assays for lipid specificity and curvature sensitivity was performed as previously described [2]. Briefly, proteins were incubated together with liposomes followed by centrifugation and analysis of the pellet and supernatant by SDS-PAGE and Coomassie staining. *In vitro* liposome tubulation assays were performed and analysed as previously described [2].

Isothermal titration calorimetry

The binding of synthetic peptides from Dynamin1 to purified GRAF1 SH3 domain was measured by isothermal titration calorimetry (ITC) using a VP-ITC (MicroCal Inc., USA). All experiments were performed in 100mM HEPES/NaOH pH 7.4, 50mM NaCl and 2mM DTT at 10°C. Protein concentrations were determined by absorbance at 280nm. 1.36ml of 51μM GRAF1 SH3 domain was loaded into the cell. The peptides (at 1mM which were custom-designed and manufactured at the Institute of Biomolecular Sciences, University of Southampton, UK) were injected from a syringe in 5μl steps every 3.5 minutes. The heat of the dilution of the ligand was subtracted from the data prior to fitting. Titration curves were fitted to the data using the ORIGIN program (MicroCal Inc.) which yielded the stoichiometry (~1), the binary association constant K_a ($=K_d^{-1}$) and the enthalpy of binding. The entropy of binding ($\Delta S°$) was calculated from the relationship $\Delta G° = - RT \bullet \ln(K_a)$ and the Gibbs-Helmholtz equation.

Cell culture and transfections

HeLa cells were grown in RPMI 1640 or MEM media (GIBCO) supplemented with LGlutamine, 10% foetal bovine serum, non-essential amino acids (for MEM), and transfected using Genejuice (Novagen) for transient protein expression. For primary cultures, rat hippocampal neurons/astrocytes were prepared by trypsin digestion and mechanical trituration from E18 or P1 Sprague-Dawley rats and plated onto poly L-lysine coated coverslips. Cells were cultured in B27-supplemented Neurobasal media. For GRAF1 depletion, HeLa cells were transfected with Stealth siRNAs specific against human GRAF1 using Lipofectamine 2000 (Invitrogen) according to manufacturers instructions. The Invitrogen siRNA duplex sequences used were siRNAa (UUA UCU CCC AUU CAG CAC AGA UAU C/ GAU AUC UGU GCU GAA UGG GAG AUA A), and siRNAb (UUU GAA ACU GGU ACA UCA UGA GUG G/CCA CUC AUG AUG UAC CAG UUU CAA A). Cells were cultured for an additional 48 hours for efficient silencing of the GRAF1 expression. Stealth Block-it siRNA (Invitrogen) was used as a control. AP2 siRNA was used as previously described [3]. Caveolin-1 knock-out mouse embryonic fibroblasts (KO MEFs) were generated and grown as previously described [4]. NIH 3T3 cells were cultured as per ECACC guidelines.

Trafficking assays

For immunofluorescence trafficking assays, biotinylated holo-transferrin, (Sigma Aldrich), Alexa Fluor 647-conjugated transferrin (Invitrogen), Alexa Fluor 546/555- conjugated CTxB (Invitrogen), DiI (Invitrogen), FITC-dextran (10kDa MW, used for fluorimetric uptake assay, Invitrogen), and biotinylated dextran (10kDa MW, used for immunofluorescent uptake assays, Invitrogen), were diluted in pre-warmed media, added to cells and incubated for time periods and temperatures as described in figure legends. After washing, cells were fixed and subjected to

immunofluorescence analysis as described below. For quantitative analysis of dextran endocytosis, HeLa cells in 35mm dishes were transfected with siRNAs/control siRNAs 48 hours prior to the experiment. Fluorescein isothiocyanate (FITC)-dextran (Sigma-Aldrich) was diluted in media to a concentration of 1mg/ml and added to cells before incubation for 15 minutes at the indicated temperature. Cells were washed twice in media and once in PBS before lysis in 1% NP- 40 in PBS supplemented with protease inhibitors. The lysate was centrifuged at 20,000g for 20 minutes at 4°C and the protein concentration in the supernatant was measured using the BCA Protein Assay Kit (Pierce) for normalization. The amount of FITC-dextran in the supernatant was measured as the emission at 515nm after exciting at 488nm using a FP- 6500 spectrofluorometer with Spectra Manager software (JASCO). The MHC Class I uptake assay was performed according to reference [5]. Briefly, HeLa cells transiently transfected with GFP-tagged GRAF1 or GRAF1 BAR+PH for 16 hours were incubated with W6/32 anti-MHC Class I antibody (American Type Culture Collection) diluted in culture media for 5 or 15 minutes at 37°C to allow endocytosis. Cells were washed in PBS and surface-bound antibody was removed by a 30 second acid wash followed by a wash in culture media to re-adjust the pH. Cells were fixed and MHC Class I was visualised using Alexa568-conjugated antibodies.

Fixed sample and real time imaging

For immunofluorescence analysis, HeLa cells were fixed in 3% paraformaldehyde in phosphate-buffered saline (PBS) for 15 minutes at 37°C (to preserve intracellular tubules which are disrupted by fixation at lower temperatures), or 4°C (to demonstrate this temperature dependence), then washed and blocked in 5% goat serum, with 0.1% saponin, in PBS before staining with the appropriate antibodies in 1% goat serum, 0.1% saponin in PBS using standard protocols. Confocal images were taken sequentially using a BioRad Radiance system and LaserSharp software (BioRad). Epifluorescence images were taken using a Zeiss Axioimager Z1 system with AxioVision software. For real time microscopy of the dynamics of GRAF1- and GRAF1 BAR+PH- positive tubules, transfected cells on glass-bottom Petri dishes (WillCo Wells BV, Amsterdam) were washed with buffer (125mM NaCl, 5mM KCl, 10mM D-glucose, 1mM $MgCl_2$, 2mM $CaCl_2$ and 25mM HEPES) and images were taken using a 5-live scanning microscope (Zeiss) or spinning disc confocal system (Improvision) with subsequent analysis in LSM Image Browser (Zeiss), ImageJ (freeware) or Volocity (Improvision). Caveolin1-KO MEFs and control MEFs grown on 12mm coverslips were cotransfected with GFP-GPI and myc-tagged GRAF1 or GRAF1 BAR+PH. Anti-GFP was bound to cells on ice for 30 minutes in unsupplemented CO_2-independent medium (Gibco). Cells were washed in CO_2-independent medium to remove unbound antibody prior to internalization in pre-warmed growth media (10% foetal bovine serum (Cambrix), 2mM L-glutamine in DMEM (Gibco)) at 37°C, 5% CO_2 for 2, 10 or 40 minutes. Post internalization, cells were washed in ice-cold CO_2-independent medium and acid stripped using 500mM glycine pH2.2 in ice cold PBS for 1 minute. To remove intensive cytosolic labeling, acid-stripped live cells were permeablized in ice-cold PBS containing 0.05% saponin (Sigma) for 5 minutes. Cells were washed 2 x 1 minute in ice cold PBS before fixation in 2% paraformaldehyde. Internalized anti-GFP was labeled with Alexa Fluor- 660-conjugated goat anti-Rabbit secondary antibody. Myc-tagged GRAF1 FL or BAR+PH was labeled with anti-myc (9B11) primary and Alexa-Fluor-555 goat-anti mouse secondary antibodies. Fluorescence microscopy was carried out using an Axiovert 200m SP LSM 510 META confocal laser-scanning microscope (Zeiss). Images were captured under oil with a 63x plan-APOCHROMAT objective, at appropriate excitation and emission wavelengths. Images were processed using Adobe

Photoshop CS2. Quantification of anti-GFP and GRAF1/GRAF1 BAR+PH colocalization was carried out using Volocity 3.7.0. In brief, target cells were cropped and blue and red channels overlaid to generate a colocalization coefficient based on a percentage of blue voxels that identify with red voxels. Background was subtracted using the automatic threshold feature. Five to seven images across three independent experiments were used to calculate average colocalization and standard error of the mean (SEM). Relative pixel numbers of anti-GFP in each image was calculated in Adobe Photoshop CS2 based on the histogram of each image. For real-time endocytosis experiments, NIH 3T3 cells grown in 35mmM glass bottom dishes were transfected with GFP-tagged GRAF1 or GRAF1 BAR+PH constructs. Cells were washed in ice-cold CO2-independent medium before binding Alexa Fluor-555-conjugated CTxB and/or Alexa Fluor-647-conjugated transferrin for 30 minutes on ice. Appropriate cells were identified using an Axiovert 200m SP LSM 510 META confocal laser-scanning microscope in ice-cold CO2-independent medium. Medium was exchanged for prewarmed CO2-independent medium plus 10% heat-inactivated Serum Supreme and images captured using Lasersharp 2000 4.0. Frames were captured every 10 seconds for 50-100 frames. Images were processed using Image J v1.37 and converted to Quicktime files.

Supplemental References

1. Praefcke, G.J., and McMahon, H.T. (2004). The Dynamin superfamily: universal membrane tubulation and fission molecules? Nat. Rev. Mol. Cell Biol. *5*, 133-147.
2. Peter, B.J., Kent, H.M., Mills, I.G., Vallis, Y., Butler, P.J., Evans, P.R., and McMahon, H.T. (2004). BAR domains as sensors of membrane curvature: the amphiphysin BAR structure. Science *303*, 495-499.
3. Motley, A., Bright, N.A., Seaman, M.N., and Robinson, M.S. (2003). Clathrin-mediated endocytosis in AP-2-depleted cells. J. Cell Biol. *162*, 909-918.
4. Kirkham, M., Fujita, A., Chadda, R., Nixon, S.J., Kurzchalia, T.V., Sharma, D.K., Pagano, R.E., Hancock, J.F., Mayor, S., and Parton, R.G. (2005). Ultrastructural identification of uncoated caveolin-independent early endocytic vehicles. J. Cell Biol. *168*, 465-476.
5. Caplan, S., Naslavsky, N., Hartnell, L.M., Lodge, R., Polishchuk, R.S., Donaldson, J.G., and Bonifacino, J.S. (2002). A tubular EHD1-containing compartment involved in the recycling of major histocompatibility complex class I molecules to the plasma membrane. EMBO J. *21*, 2557-2567.

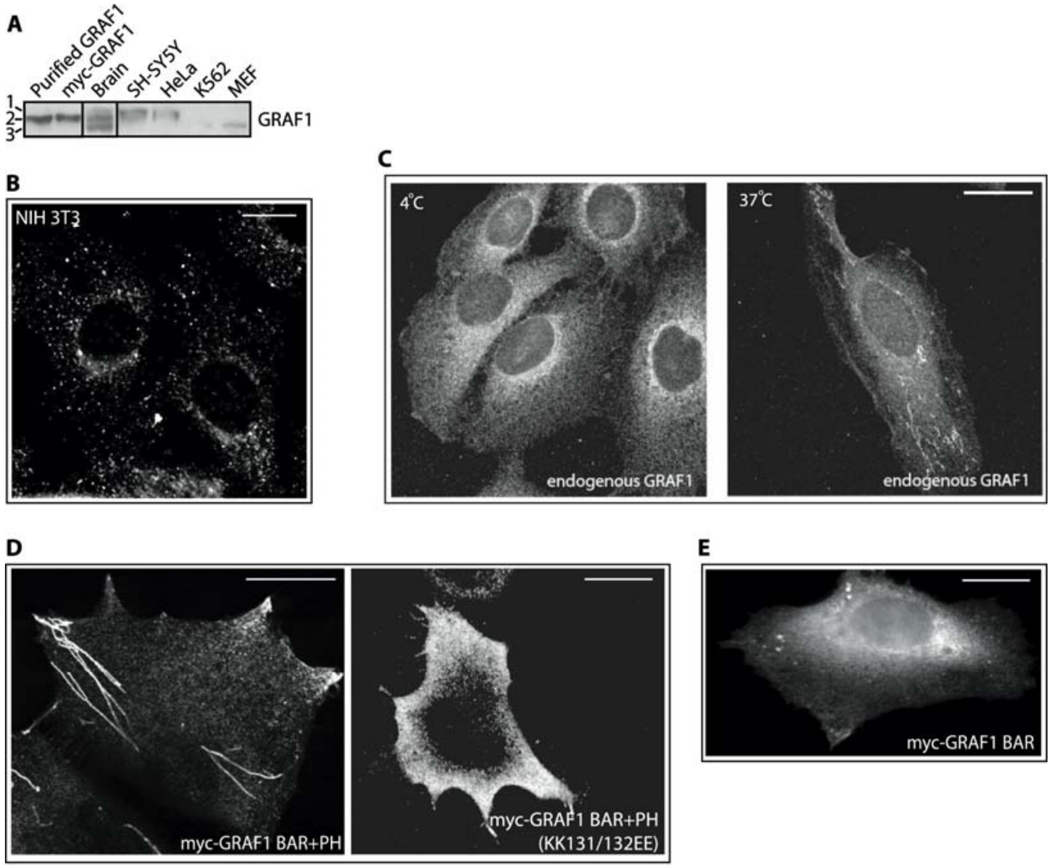

Figure S1 | Localisation of GRAF1 to tubular membrane structures is temperature sensitive and dependent on the BAR and PH domains. A, Western blots showing the different forms of GRAF1 detected in adult rat brain and their differential presence/absence in cultured SH-SY5Y (human neuroblastoma), HeLa (human fibroblast), K562 (human Chronic Myeloid Leukaemia), and MEF (mouse embryonic fibroblast), cells. Western blots of purified GRAF1 and myc-tagged GRAF1 (from lysates of HeLa cells overexpressing this protein) are shown for comparison. **B,** Confocal micrograph of an NIH 3T3 cell stained for endogenous GRAF1 distribution. **C,** Confocal micrographs of HeLa cells fixed either at 4°C or 37°C for 10 minutes in 4% paraformaldehyde and then stained for endogenous GRAF1. Note the absence of GRAF1-positive tubules in the 4°C fixation image. **D**, Confocal micrographs showing the tubular localization of overexpressed myc-tagged GRAF1 BAR+PH protein in HeLa cells and the cytoplasmic localization of a similarly overexpressed protein with a BAR domain mutation (KK131/132EE). **E,** Confocal micrograph showing the cytoplasmic and punctate localization of overexpressed myc-tagged GRAF1 BAR protein in HeLa cells. **Scale bars = 10μm.**

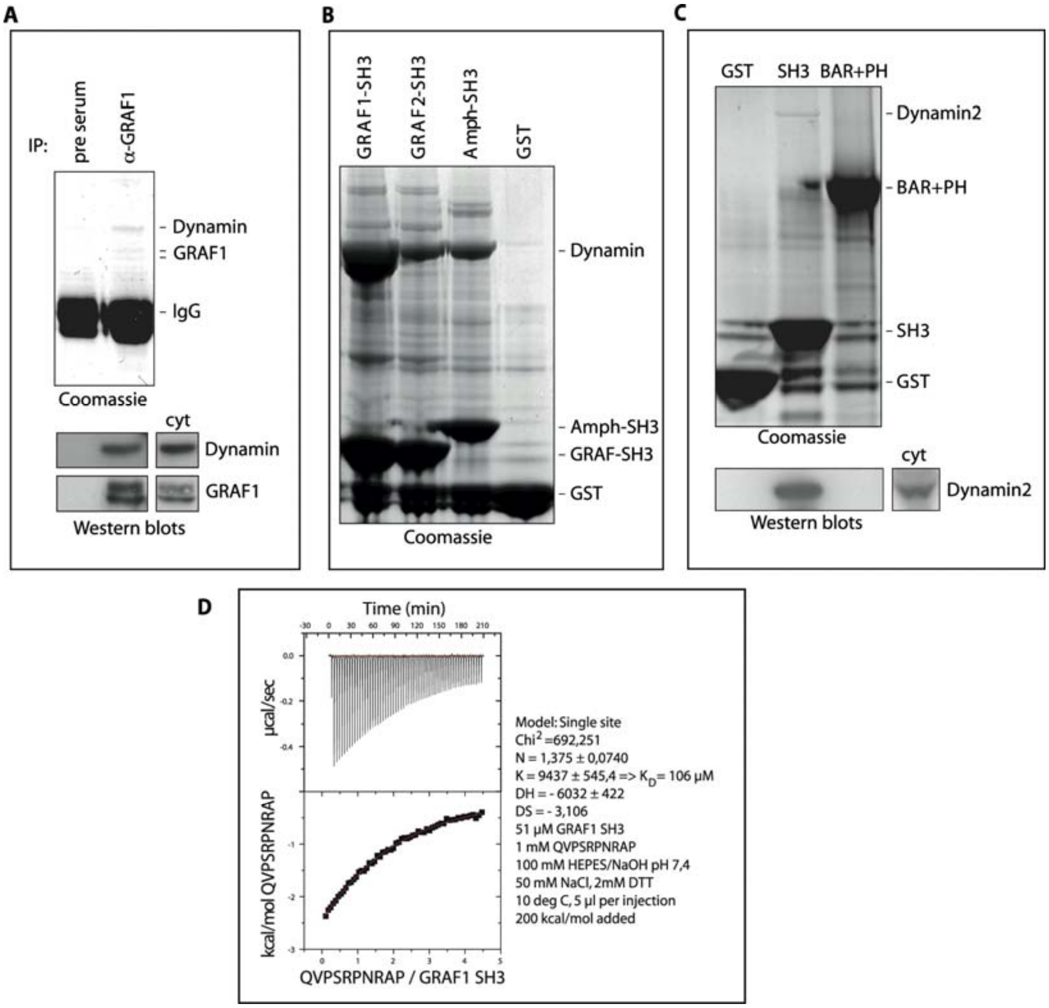

Figure S2 A-D| Supplementary biochemical data on the binding between GRAF1 and Dynamin. A, Coomassie-stained gel and confirmatory Western blots of co-immunoprecipitation experiments in rat brain cytosol performed with either control pre-immunization serum (pre-serum) or the Ab3 antibody. Bands in the Coomassie-stained gel were identified by mass spectrometry as described. **B and C,** Coomassie-stained gel and Western blots of pull-down experiments from mouse brain lysate (B) or HeLa cell cytosol (C) with beads bound to GST (control) or GST-tagged GRAF1 BAR+PH, or SH3 proteins. The bands in the Coomassie-stained gel were identified by mass spectrometry as described. Note the major band of Dynamin present in the SH3 lanes, which is not present in the control or BAR+PH condition. 'cyt' marks the HeLa cell lysate (positive control) lane. **D,** The upper panel shows a raw trace from isothermal titration calorimetry performed as described. The lower panel shows the fitting of this data to a one-site binding model from which the affinity (shown) can be calculated. GRAF1 SH3 domain and peptide concentrations, as well as injection volumes and times are shown.

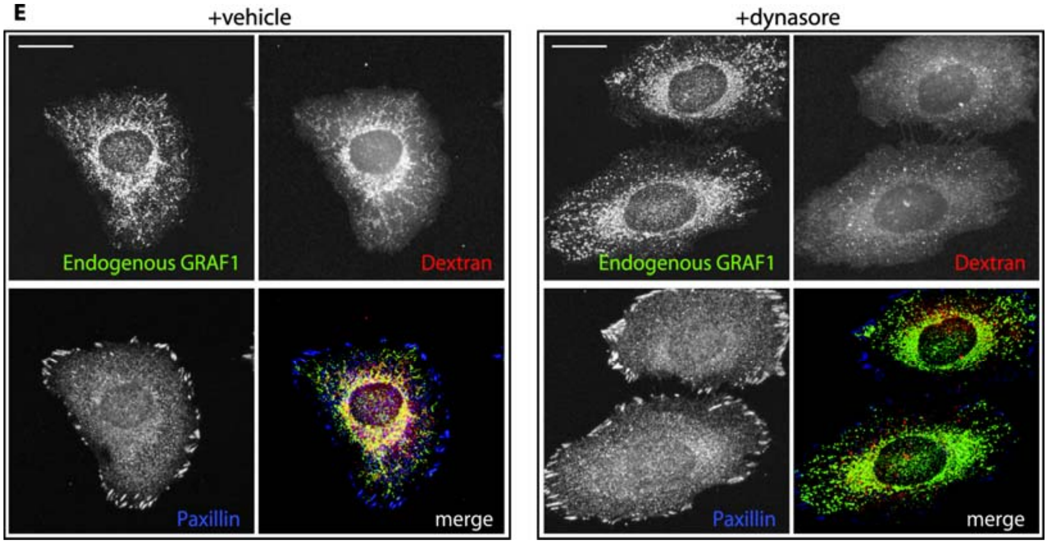

Figure S2 E | Dynasore inhibits the uptake of dextran and affects the localization of GRAF1. E, Confocal micrographs (maximum projections) of HeLa cells treated with either DMSO (vehicle) or 100μM dynasore for 1 hour before addition of dextran for 15 minutes, fixation, and immunostaining for dextran and the focal adhesion marker paxillin. **Scale bars = 10μm.**

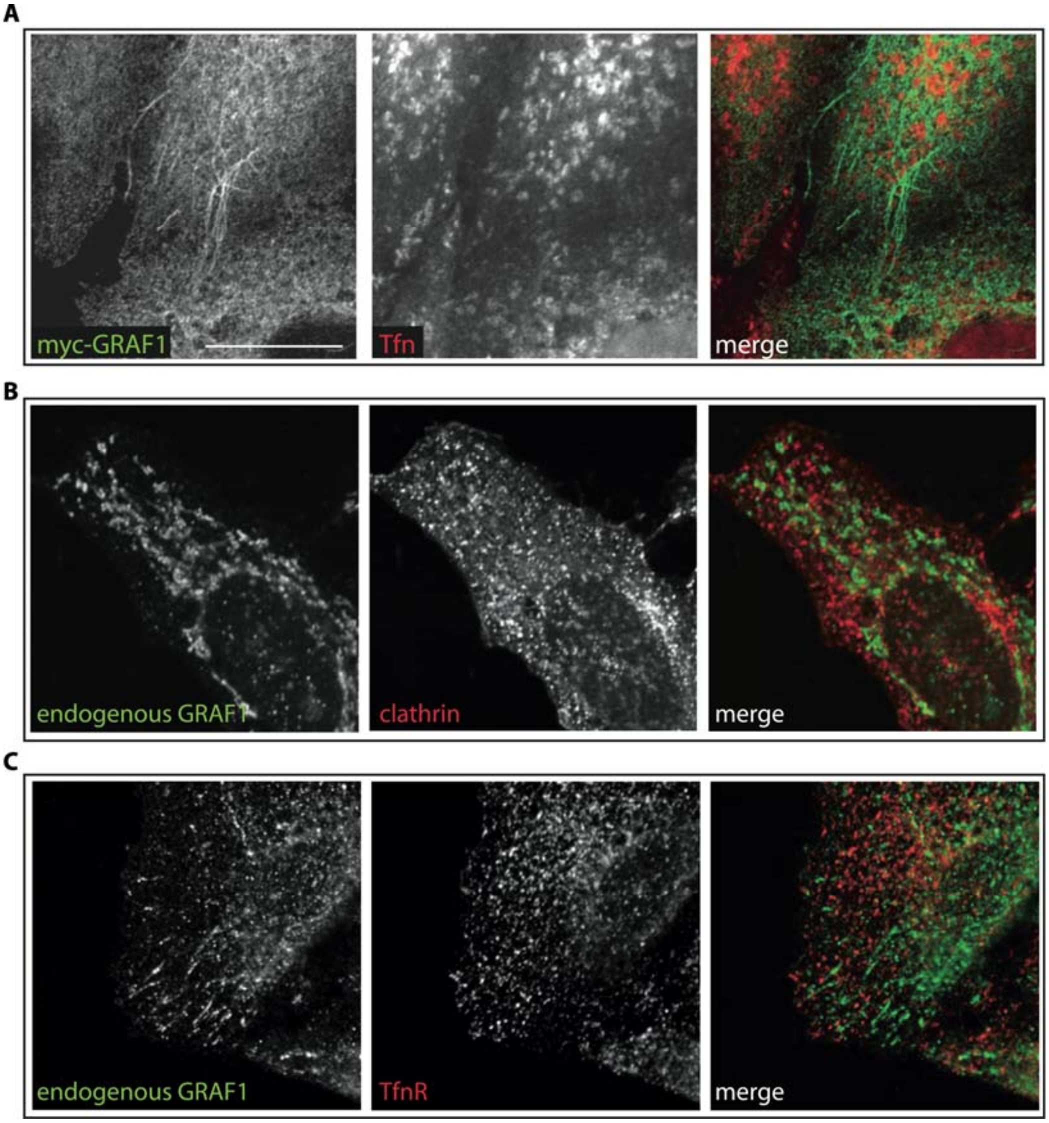

Figure S3 | GRAF1-positive endocytic structures are Clathrin-independent and exclude transferrin A-C, Confocal fluorescent micrographs of HeLa cells stained for endogenous GRAF1 and Clathrin (A), transferrin (B) or transferrin receptor (B). The depicted images are used to show some of the different morphologies of GRAF1-positive structures that are observed in these cells. **Scale bars = 10μm.**

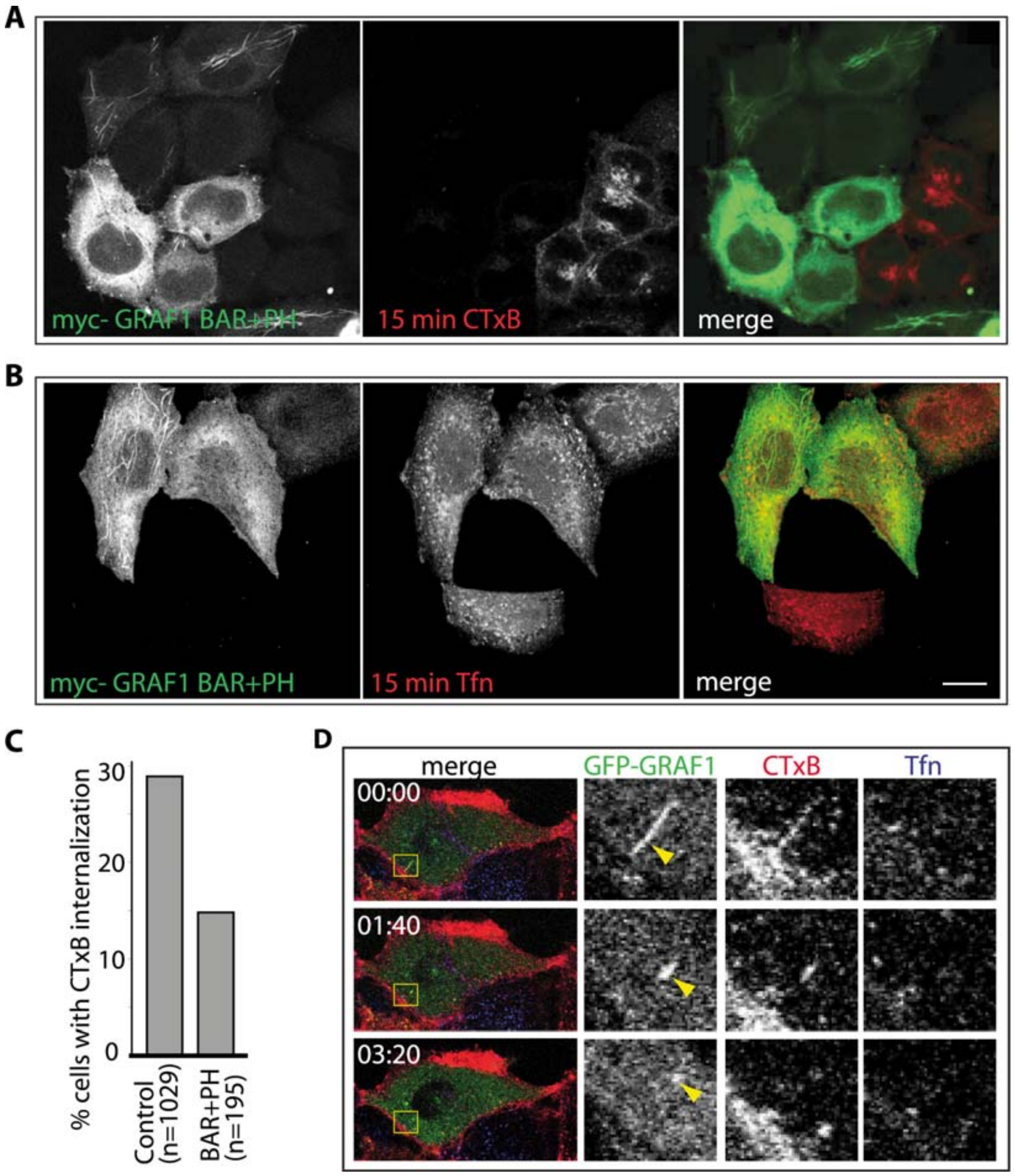

Figure S4 | GRAF1 BAR+PH overexpression affects CTxB uptake but not transferrin uptake. A and B, Confocal micrographs of HeLa cells transfected with myc-tagged GRAF1 BAR+PH and incubated with CTxB or transferrin for 15 minutes before fixation and staining. **C,** The graph shows the quantification of images such as depicted in (A). Cells were scored for expression of GRAF1 BAR+PH (over a threshold corresponding to maximum autofluorescence) and CTxB internalization (over an arbitrarily-set threshold above background). Note the reduction of the number of transfected cells internalising CTxB compared with controls. **D,** Live cell microscopy of NIH 3T3 cells expressing GFP-tagged GRAF1 and incubated with CTxB and transferrin (Tfn) at 4°C before chasing their internalization from the time of warming to 37°C (time=00:00). Note the internalising GRAF1-positive tubule containing CTxB. Note also the lack of colocalization of GRAF1-positive tubules with internalized transferrin. Time is given as minutes:seconds. This sequence is taken from Movie S3. **Scale bars = 10μm.**

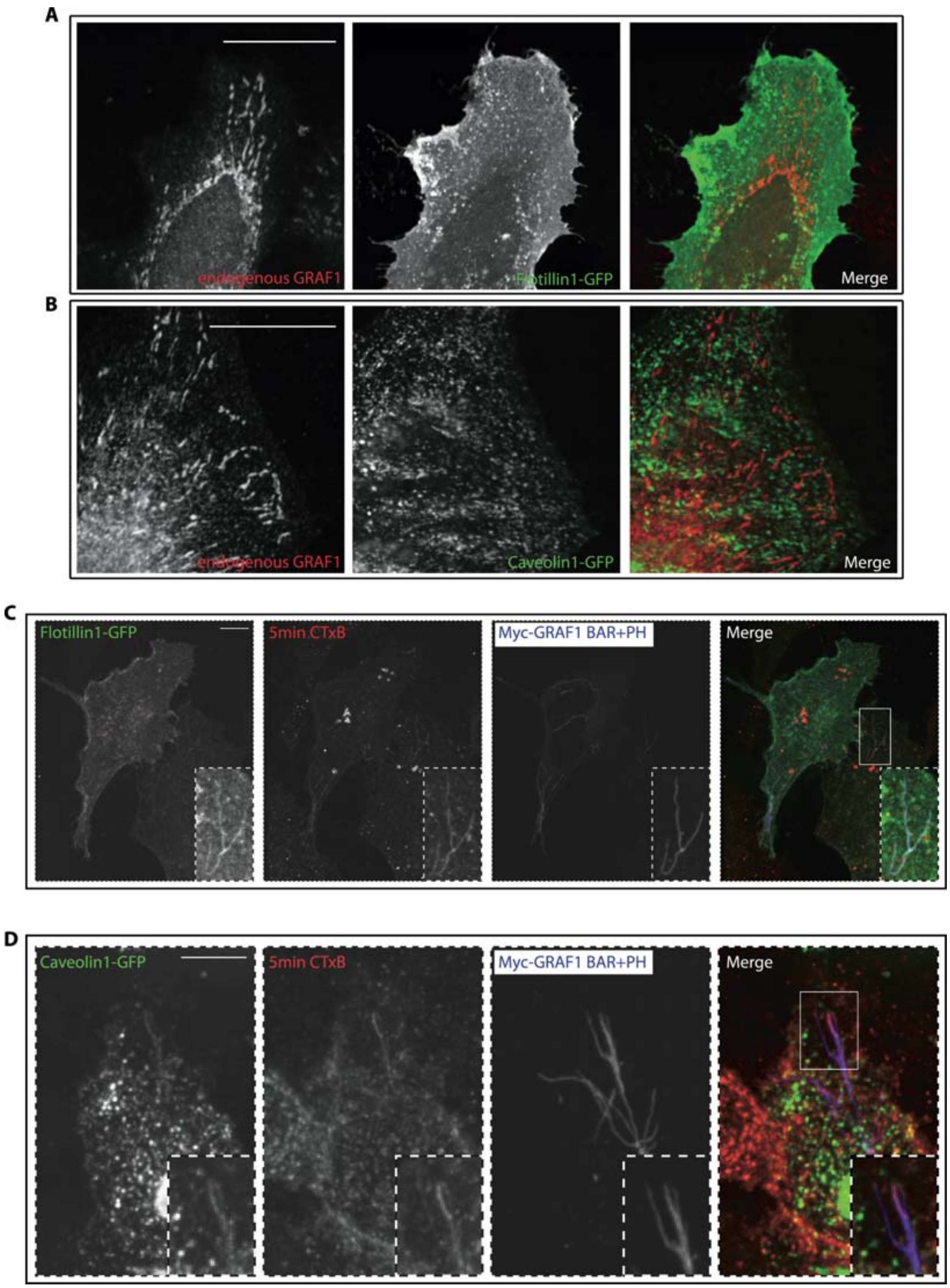

Figure S5 | Nature of GRAF1-positive endocytic structures. A and B, Confocal micrographs of HeLa cells overexpressing GFP-tagged flotillin1 (A) or GFP-tagged caveolin1 (B) and co-stained for endogenous GRAF1. Note the lack of colocalization. **C and D,** Confocal micrographs of HeLa cells overexpressing myc-tagged GRAF1 BAR+PH and flotillin1 (E) or caveolin1 (F) incubated with CTxB for 5 minutes. Note the colocalization of GRAF1 BAR+PH and flotillin1 in CTxB-positive tubular structures. **Scale bars = 10µm.**

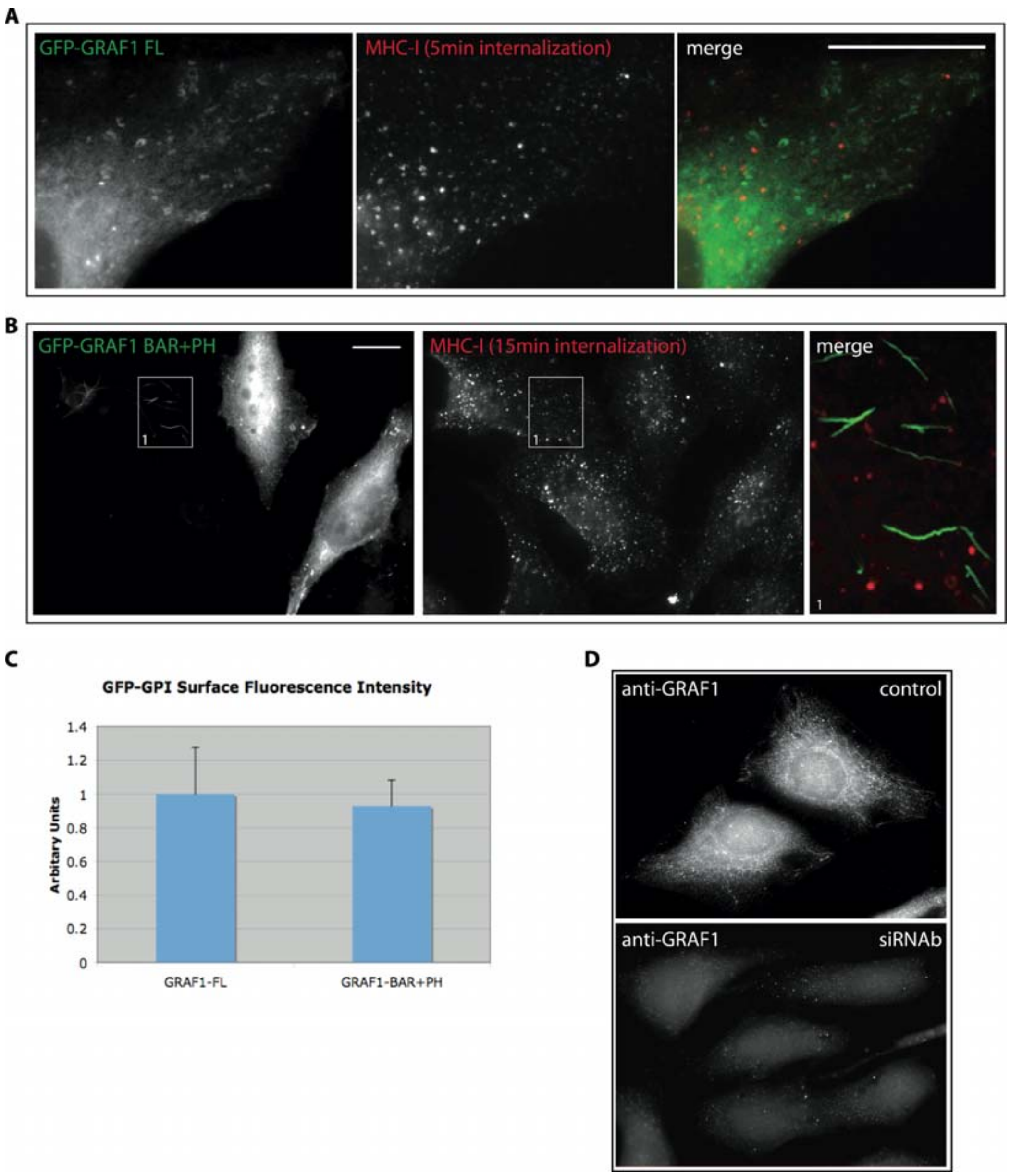

Figure S6 | GRAF1 BAR+PH overexpression does not affect the uptake of MHC Class I which enter GRAF1-negative compartments. A and B, Epifluorescence micrographs of HeLa cells, transiently transfected with GFP-tagged GRAF1 FL (A) or GRAF BAR+PH (B) were pulsed with anti-MHC Class I antibody for 5 (A) or 15 minutes (B) at 37°C followed by a brief acid wash to remove surface-bound antibody. MHC Class I was visualized using Alexa568-conjugated secondary antibodies. **C,** Quantitation of surface GFP-GPI levels in cells overexpressing this protein with myc-tagged GRAF1 FL or GRAF1 BAR+PH. **D,** Confocal micrographs of HeLa cells treated with siRNA against GRAF1 or a control siRNA and stained for endocgenous GRAF1. **Scale bars = 10μm.**
