# Supplementary figures and images for: The GTPase-Activating Protein GRAF1 Regulates the CLIC/GEEC Endocytic Pathway
Source: Curr Biol. 2008 Nov 25;18(22-2):1802–8. doi: 10.1016/j.cub.2008.10.044 (PMC2726289; doi:10.1016/j.cub.2008.10.044)

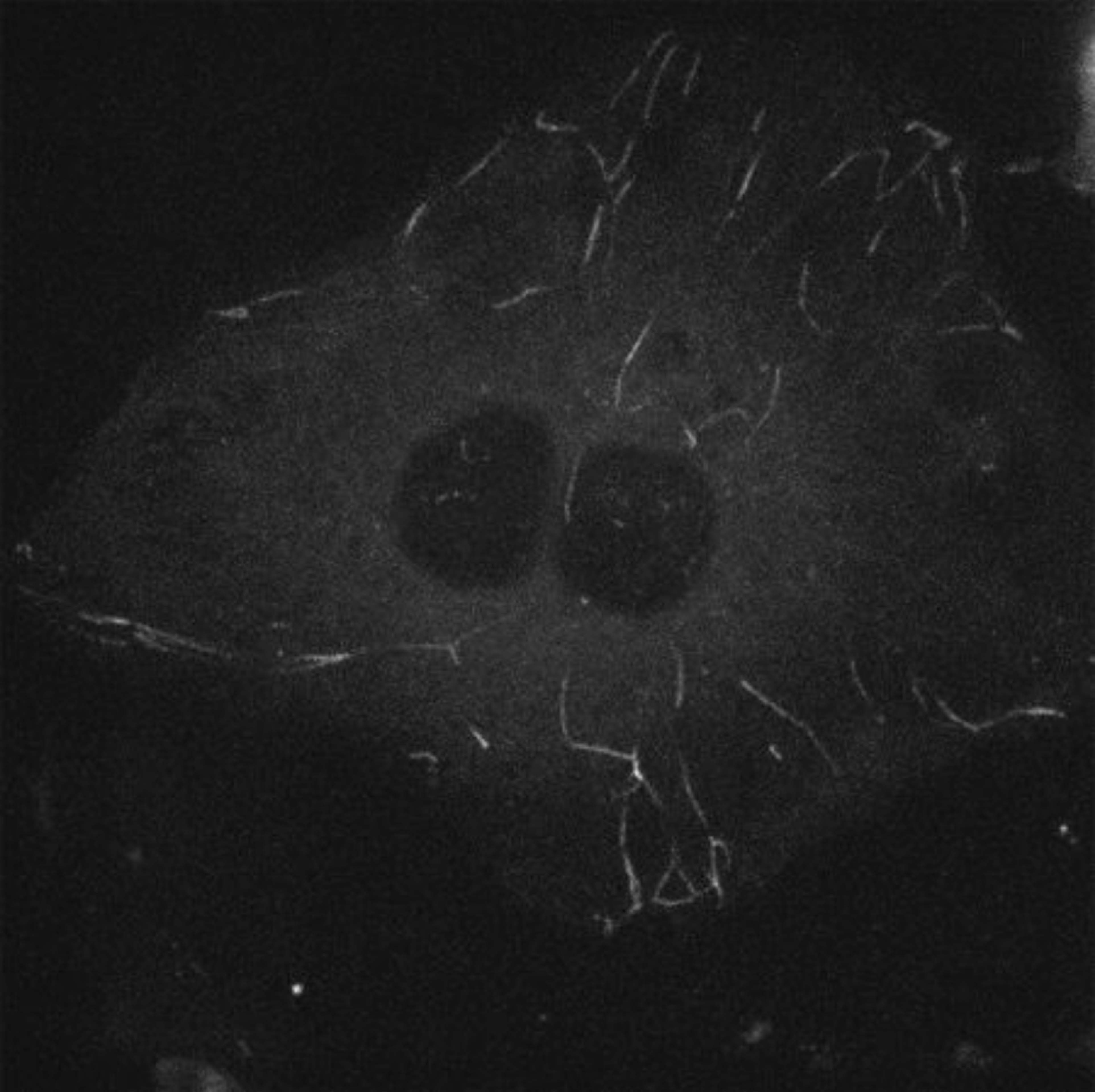

Supplement: Movie S1. GRAF1-Positive Tubules Are Highly Dynamic — Movie of HeLa cell overexpressing GFP-tagged GRAF1, manipulations of which are shown in Figures 2A and 2B. Movie speed is 40 times real time. [file mmc2.jpg]

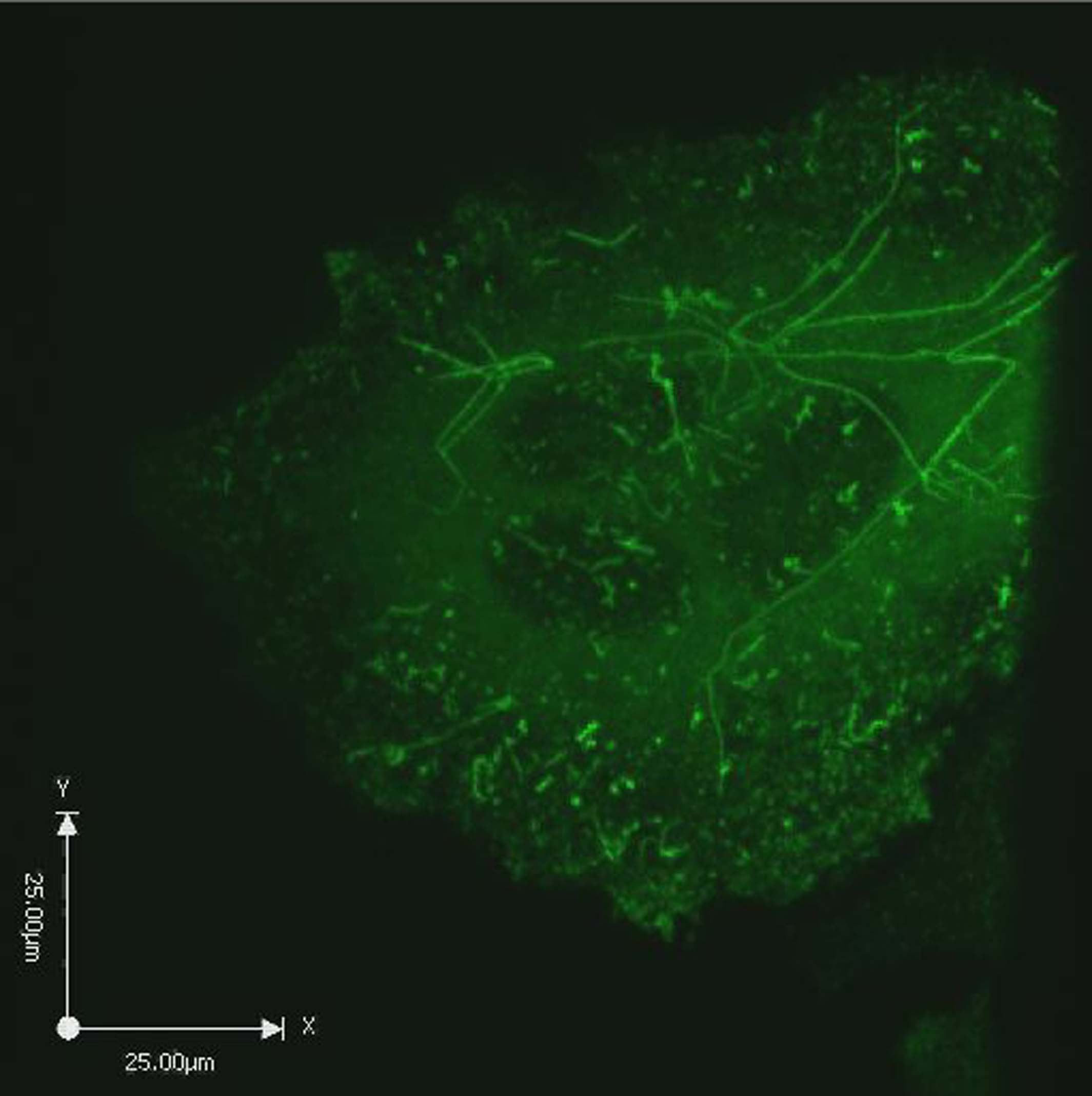

Supplement: Movie S2. GRAF1 BAR+PH-Positive Tubules Are Relatively Static — Movie of HeLa cell overexpressing GFP-tagged GRAF1 BAR+PH, a manipulation of which is shown in Figure 2D. Movie speed is 40 times real time. [file mmc3.jpg]

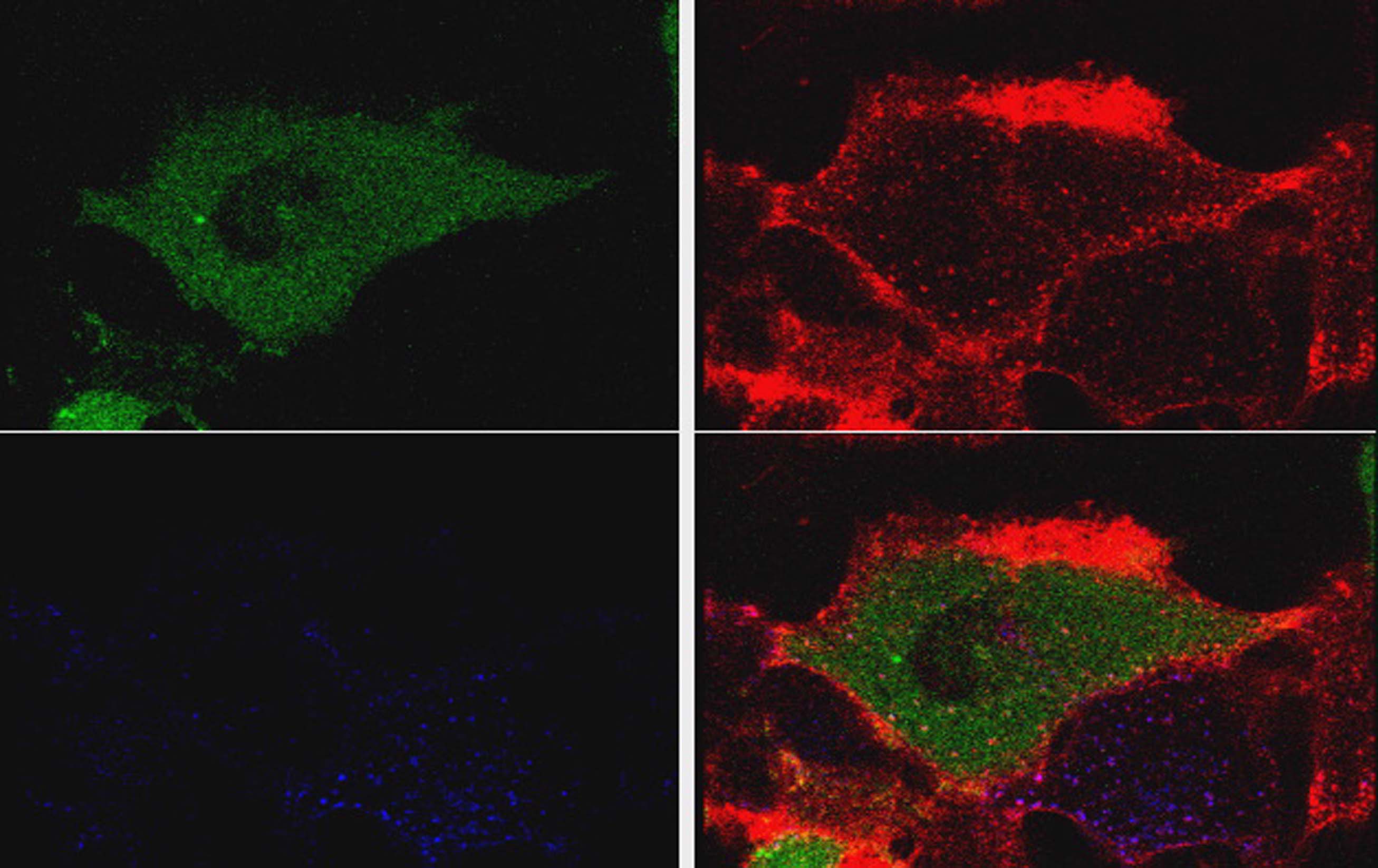

Supplement: Movies S3. CTxB Internalization Occurs into GRAF1-Positive Tubules — Movie of NIH 3T3 cells overexpressing GFP-tagged GRAF1 (stills of which are shown in Figure 6D) and incubated with CTxB as described. Movie speed is 80 times real time. [file mmc4.jpg]
